# Oral administration of the K_ATP _channel opener diazoxide ameliorates disease progression in a murine model of multiple sclerosis

**DOI:** 10.1186/1742-2094-8-149

**Published:** 2011-11-02

**Authors:** Noemí Virgili, Juan F Espinosa-Parrilla, Pilar Mancera, Andrea Pastén-Zamorano, Javier Gimeno-Bayon, Manuel J Rodríguez, Nicole Mahy, Marco Pugliese

**Affiliations:** 1 Neurotec Pharma SL, Bioincubadora PCB-Santander, Parc Científic de Barcelona, c/Josep Samitier 1-5, 08028 Barcelona, Spain; 2 Unitat de Bioquímica i Biologia Molecular, Departament de Ciències Fisiològiques I, Facultat de Medicina, Institut d'Investigacions Biomèdiques August Pi i Sunyer (IDIBAPS) and Centro de Investigación Biomédica en Red sobre Enfermedades Neurodegenerativas (CIBERNED), c/Casanova 143, 08036 Barcelona, Spain

**Keywords:** Diazoxide, experimental autoimmune encephalomyelitis, K_ATP _channel, microglia, multiple sclerosis, neuroprotection

## Abstract

**Background:**

Multiple Sclerosis (MS) is an acquired inflammatory demyelinating disorder of the central nervous system (CNS) and is the leading cause of nontraumatic disability among young adults. Activated microglial cells are important effectors of demyelination and neurodegeneration, by secreting cytokines and others neurotoxic agents. Previous studies have demonstrated that microglia expresses ATP-sensitive potassium (K_ATP_) channels and its pharmacological activation can provide neuroprotective and anti-inflammatory effects. In this study, we have examined the effect of oral administration of K_ATP _channel opener diazoxide on induced experimental autoimmune encephalomyelitis (EAE), a mouse model of MS.

**Methods:**

Anti-inflammatory effects of diazoxide were studied on lipopolysaccharide (LPS) and interferon gamma (IFNγ)-activated microglial cells. EAE was induced in C57BL/6J mice by immunization with myelin oligodendrocyte glycoprotein peptide (MOG_35-55_). Mice were orally treated daily with diazoxide or vehicle for 15 days from the day of EAE symptom onset. Treatment starting at the same time as immunization was also assayed. Clinical signs of EAE were monitored and histological studies were performed to analyze tissue damage, demyelination, glial reactivity, axonal loss, neuronal preservation and lymphocyte infiltration.

**Results:**

Diazoxide inhibited *in vitro *nitric oxide (NO), tumor necrosis factor alpha (TNF-α) and interleukin-6 (IL-6) production and inducible nitric oxide synthase (iNOS) expression by activated microglia without affecting cyclooxygenase-2 (COX-2) expression and phagocytosis. Oral treatment of mice with diazoxide ameliorated EAE clinical signs but did not prevent disease. Histological analysis demonstrated that diazoxide elicited a significant reduction in myelin and axonal loss accompanied by a decrease in glial activation and neuronal damage. Diazoxide did not affect the number of infiltrating lymphocytes positive for CD3 and CD20 in the spinal cord.

**Conclusion:**

Taken together, these results demonstrate novel actions of diazoxide as an anti-inflammatory agent, which might contribute to its beneficial effects on EAE through neuroprotection. Treatment with this widely used and well-tolerated drug may be a useful therapeutic intervention in ameliorating MS disease.

## Background

Multiple Sclerosis (MS) is a chronic autoimmune, inflammatory and degenerative disease of the central nervous system (CNS) that causes significant disability. Current drugs improve the course of the disease but with limited efficacy, serious side effects and inconvenient routes of administration. For these reasons, there is a need to develop more efficacious drugs (targeting inflammation and also neurodegeneration) that are safer (avoiding life-threatening adverse events, fatal infections or cancer), have non-serious adverse events that impair quality of life (e.g., flu-like symptoms), can be administered orally and have a good profile for eventual combination therapy.

Microglial cells, the resident macrophage populations in the CNS, sustain and propagate inflammation within the CNS through antigen and/or cytokine/chemokine secretion, which are important effectors of the demyelination and neurodegeneration described in MS [1]. Perivascular microglia act as antigen-presenting cells to myelin-specific T cells and promote the CNS-confined inflammatory process. Once the process is initiated, parenchymal microglial cells are activated and elicit myelin damage and neurodegeneration by secreting pro-inflammatory and neurotoxic factors such as tumor necrosis factor alpha (TNF-α), prostaglandins, interleukin-6 (IL-6), nitric oxide (NO) or reactive oxygen species (ROS) [2,3]. Thus, microglial cells are a potential therapeutic target in inflammatory CNS disorders such as MS.

Potassium (K^+^) channel modulation is widely pursued as novel pharmaceutical strategy for the treatment of neurological disorders and autoimmune diseases [4]. In MS, activation on T cells depends on K^+ ^channel and selective targeting of two-pore domain K^+ ^channels (K_2P_5.1), voltage-gated K^+ ^channel K_V_1.3 and calcium-activated K^+ ^channel IKCa1 have been proposed for the treatment of CNS inflammation and degeneration [5-7]. ATP-sensitive K^+ ^(K_ATP_) channels are large hetero-octameric complexes consisting of four pore-forming inward-rectifying K^+ ^subunits (Kir6.x) and four regulatory sulfonylurea receptor (SURx) subunits [8]. They are considered metabolic sensors that couple cellular energy metabolism to membrane excitability by regulating potassium flux. These channels act as energy sensors of ATP production and are believed to regulate various physiological functions, such as muscle contraction and insulin secretion, by coupling cell metabolism to membrane potential [9-11]. K_ATP _channels are also present at the mitochondrial inner membrane (mito-K_ATP_) and they participate in the regulation of mitochondrial volume and membrane potential. Furthermore, their activity is related to electronic transport, metabolic energy, ROS production and mitochondrial welfare [12,13]. K_ATP _channels are found in a range of tissues and they are also widely expressed in various brain regions, where they couple electrical activity of the neuron to its metabolic state, and modulate neuronal excitability in different physiological and pathological conditions [14-16].

We previously reported that activated microglia in a rat model of neurodegeneration and in postmortem samples of patients with Alzheimer's disease (AD) strongly expressed K_ATP _channel SUR components similar to those in neurons and pancreatic beta-cells [17]. In this context, controlling the extent of microglial activation and neuroinflammation may offer prospective clinical therapeutic benefits for inflammation-related neurodegenerative disorders. Other authors have documented that pharmacological activation of K_ATP _channels can exert neuroprotective and anti-inflammatory effects on the brain against ischemia, trauma and neurotoxicants [18-21]. Therefore, the expression of K_ATP _channels by activated microglia indicates that K_ATP _channel openers (KCOs), such as diazoxide, could be used as therapeutic agents to treat inflammatory and neurodegenerative diseases like MS.

Diazoxide (7-chloro-3-methyl-4H-1,2,4-benzothiadiazine 1,1-dioxide) is a well-known small molecule that activates K_ATP _channels in the smooth muscle of blood vessels and pancreatic beta-cells by increasing membrane permeability to potassium ions. It is structurally related to the thiazide diuretics, but does not possess any discernible diuretic activity. Its binding site is located on other regions of the SUR protein than the site for other KCOs and binding with similar affinities to SUR1 and SUR2B [22]. Diazoxide-induced hyperpolarization of cell membranes prevents calcium entry via voltage-gated Ca^2+ ^channels (VGCCs), resulting in vasorelaxation and the inhibition of insulin secretion [23,24]. As a consequence, diazoxide increases the concentration of plasma glucose and produces a fall in blood pressure by a vasodilator effect on the arterioles and a reduction in peripheral resistance. Due to these actions, diazoxide has been approved and used since the 1970s for treating malignant hypertension and hypoglycemia in different European countries, the United States and Canada [25,26].

Others authors found that diazoxide-mediated cytoprotection is independent of the conductance of the mito-K_ATP _channel inhibiting succinate oxidation and succinate dehydrogenase activity [27]. These data implicate a direct mitochondrial respiratory inhibition-triggered ROS signaling mechanism in the protection of tissues by diazoxide [28].

The aims of the present study were to: (a) analyze the expression of K_ATP _channels on microglial cells and whether its pharmacological activation by diazoxide modulates the release of inflammatory mediators, and (b) study the effects of diazoxide oral administration on myelin oligodendrocyte glycoprotein peptide (MOG_35-55_)-induced experimental autoimmune encephalomyelitis (EAE), a murine model of MS.

## Methods

### Primary cell culture and cell line

The mouse microglial cell line BV-2 was purchased at the Istituto Nazionale per la Ricerca sul Cancro (IST, Genova, Italy), while primary glial cultures were obtained from 2- to 4-day old C57BL/6J mice as described previously by Saura *et al*. [29].

### Mice

Female C57BL/6J mice, 8 to 10 weeks of age, were purchased from Charles River (Sulzfeld, Germany) and maintained on a 12:12 h light:dark cycle, with standard chow and water freely available. Animals were handled according to European legislation (86/609/EEC) and all manipulations were performed in accordance with European legislation (86/609/EEC). All efforts were made to minimize the number of animals and their suffering during the experiments, and procedures were approved by the Ethics Committee of the University of Barcelona under the supervision of the Generalitat of Catalunya, Spain.

### Reagents

Diazoxide was purchased from Sigma-Aldrich (St. Louis, MO, USA). Stock solutions (50 mM) of diazoxide were prepared in dimethyl sulfoxide (DMSO, Sigma-Aldrich). Solutions for cell treatment were prepared by diluting stock solutions in culture media immediately before being added to the cells (DMSO concentration: 0.5%). Solutions for animal treatment were prepared by diluting stock solution in water every day of the treatment (DMSO concentration: 0.3%).

### Cell culture and treatment

For primary mixed glial cultures, cells were seeded at a density of 4 × 10^5 ^cells/mL and cultured in Dulbecco's modified Eagle medium-F-12 nutrient mixture supplemented with 10% heat-inactivated fetal bovine serum (FBS), 0.1% penicillin-streptomycin and 0.5 μg/mL amphotericin B (Fungizone^®^) (all from Gibco Invitrogen, Paisley, Scotland, UK). Cells were maintained at 37°C in a 5% CO_2 _humidified atmosphere. Medium was replaced every 7 days. After 19 to 21 days *in vitro *(DIV), microglia were isolated as described by Saura and collaborators [29]. Cultures obtained following this method contained > 98% of microglia. The following day, mixed glial and microglial cultures were treated with different concentrations of diazoxide 30 min before stimulation with lipopolysaccharide (LPS) (*E. coli *serotype 026:B6) 100 ng/mL and recombinant mouse interferon gamma (IFNγ) (both from Sigma-Aldrich, St. Louis, MO, USA) 10 pg/mL. As control, unstimulated cells and unstimulated cells pretreated with highest diazoxide concentration (100 μM) were used. Both contained the same final concentration of vehicle as the compound-containing wells.

BV-2 cells were cultured in RPMI-1640 medium (Gibco Invitrogen, Paisley, Scotland, UK) supplemented with 10% FBS and 0.1% penicillin-streptomycin. Cells were maintained at 37°C in a 5% CO_2 _humidified atmosphere. BV-2 cells were seeded at a density of 5 × 10^4 ^cells/mL. The following day, cells were treated with diazoxide 30 min before stimulation with LPS 100 ng/mL and IFN-γ 50 pg/mL. Control wells contained the same final concentration of vehicle as the compound-containing wells.

Culture supernatants of BV-2 and primary cells were collected 24 h after LPS/IFN-γ stimulation and stored at -20°C until assayed for nitrites, TNF-α and IL-6 content. Cell viability after treatment was determined by the 3-(4,5-Dimethyl-2-thiazolyl)-2,5-diphenyl-2H-tetrazolium bromide (MTT) reduction method.

### Nitrite, TNF-α and IL-6 quantification

Nitrite levels were quantified by the Griess reaction. Briefly, 50 μL of culture medium was mixed in a 96-well plate with 25 μL of Griess reagent A (sulfanilamide) and 25 μL of reagent B (N-1- naphthyl ethylene -diamine). After color development (10 min at 23 to 25°C), samples were measured at 540 nm on a microplate reader (BioTek ELX800, BioTek Instruments Inc., Vermont, USA). Nitrite concentration was determined from a sodium nitrite standard curve. The amount of TNF-α and IL-6 released into the culture medium was determined using an Enzyme-linked immunosorbent assay (ELISA) kit specific for mouse TNF-α (Murine TNF-α ELISA Development Kit, Peprotech, Rocky Hill, NJ, USA) and for mouse IL-6 (Mouse IL-6 Ready-SET-Go!^®^, eBioscience, San Diego, CA, USA) according to the manufacturer's instructions.

### Immunofluorescence cell staining

BV-2 cells were activated with LPS/IFN-γ for 24 h, as described above. Then, cells were fixed with cold methanol (-20°C) for 5 minutes. Cultures were blocked in phosphate buffered saline (PBS) solution containing 10% donkey serum (Sigma-Aldrich, St. Louis, MO, USA) and 1% bovine serum albumin (BSA) (VWR International Ltd, UK) for 20 minutes. Cells were then incubated with primary antibodies anti-Kir6.1 and anti-Kir6.2 (1:300 dilution, Alomone, Jerusalem, Israel), anti-CD11b (1:500 dilution, Serotec, Oxford, England, UK) at 4°C overnight, followed by secondary antibodies Alexa^®^488 and 596 (1:500, Molecular Probes, Invitrogen, Eugene, OR, USA) for 1 h in blocking solution. Slides were mounted in ProLong Gold antifade medium (Molecular Probes, Invitrogen, Eugene, OR, USA) and images were acquired by SP1 confocal microscope (Leica Microsystems GmbH, Wetzlar, Germany), located at the Institut de Biologia Molecular de Barcelona, Microscopy Unit, Parc Científic de Barcelona, Barcelona, Spain.

### Phagocytosis assay

The phagocytic ability of microglia was determined by the uptake of 2-μm red fluorescent microspheres (Molecular Probes, Invitrogen, Eugene, OR, USA) by BV-2 cells. Cells were treated with diazoxide 100 μM and activated with LPS/IFN-γ, as described above, and then incubated with microspheres at a concentration of 0.01% for 30 min in the dark at 37°C and 5% CO_2_. Cells were rinsed twice in PBS solution, pelleted at 1,000 *g *for 5 min and resuspended in 300 μL PBS. Cells were kept on ice and analyzed by flow cytometry. The single-cell fluorescent population was selected on a forward-side scatter scattergram using an Epics XL flow cytometer (Coulter Corporation, Miami, Florida) located at Technical and Scientific Center-University of Barcelona, Parc Científic Barcelona, Barcelona, Spain.

Some samples were fixed with 3% paraformaldehyde solution and stained using FITC conjugated anti-α-tubulin antibody (Sigma-Aldrich, St. Louis, MO, USA) and Hoechst 34580 (Molecular Probes, Invitrogen, Eugene, OR, USA) nuclear staining for image acquisition.

### 3-(4,5-Dimethyl-2-thiazolyl)-2,5-diphenyl-2H-tetrazolium bromide (MTT) reduction method

MTT reduction assay was used as an indicator of cell viability. MTT (Sigma-Aldrich, St. Louis, MO, USA) was added to a well at a final concentration of 0.5 mg/mL. After MTT incubation at 37°C, DMSO was added and cells were gently resuspended. Absorbances at 560 and 620 nm were recorded with a microplate reader (BioTek ELX800, BioTek Instruments Inc., Vermont, USA).

### Isolation of total protein

For spinal cord total protein extraction, tissue (100 mg) was placed into a 1.5-mL microtube on ice containing 500 μL ice-cold RIPA extraction buffer (Sigma-Aldrich, St. Louis, MO, USA) supplemented with complete protease inhibitor cocktail tablets (Roche Diagnostics, Basel, Switzerland). The sample was homogenized with a pipette tip on ice for 30 min. The homogenate was centrifuged at 6000 *g *for 15 min at 4°C. The supernatant was separated and stored at -80°C until use. For isolation of total proteins from cell cultures, after a cold PBS wash, total proteins were recovered in 100 μL per well of RIPA buffer supplemented with complete protease inhibitor cocktail tablets. The samples were sonicated and stored at -80°C. Protein amount was determined by the Lowry assay (Total Protein Kit micro-Lowry, Sigma-Aldrich, St. Louis, MO, USA).

### Western blot

30 to 40 μg of proteins from denatured (100°C for 5 min) total extracts were subjected to sodium dodecyl sulfate-polyacrylamide gel electrophoresis together with a molecular weight marker (Full Range Rainbow Molecular Weight Marker, Amersham, Buckinghamshire, UK), and transferred to a polyvinylidene difluoride membrane (Millipore, Bedford, MA, USA). After washing in Tris-buffered saline (TBS: 20 mM Tris, 0.15 M NaCl, pH 7.5) for 5 min, dipping in methanol for 10 s and drying in air, the membranes were incubated with the following primary antibodies overnight at 4°C: polyclonal rabbit anti-Kir 6.1 or polyclonal rabbit anti-Kir 6.2 (both 1:500, Alomone, Jerusalem, Israel), polyclonal rabbit anti-inducible nitric oxide synthase (iNOS) (1:200, Millipore, Bedford, MA, USA), polyclonal rabbit anti-cyclooxygenase-2 (1:2000, Santa Cruz Biotechnology, St. Cruz, CA, USA) and monoclonal mouse anti-β-actin (1:50000, Sigma-Aldrich, St. Louis, MO, USA) diluted in immunoblot buffer (TBS containing 0.05% Tween-20 and 5% non-fat dry milk). The membranes were then washed twice in 0.05% Tween-20 in TBS for 15 s and incubated with the following horseradish peroxidase (HRP)-labeled secondary antibodies for 1 h at 23 to 25°C: donkey anti-rabbit (1:5000, Amersham, Buckinghamshire, UK) or goat anti-mouse (1:5000, Santa Cruz Biotechnology, St. Cruz, CA, USA). After extensive washes in 0.05% Tween-20 in TBS, they were incubated in ECL-Plus (Amersham, Buckinghamshire, UK) for 5 min. Membranes were then exposed to the camera and the pixel intensities of the immunoreactive bands were quantified using the percentage adjusted volume feature of Quantity One 5.6.4 software (Bio-Rad Laboratories, Hercules, CA, USA). Data are expressed as the ratio of the band intensity of the protein of interest to the loading control protein band (β-actin).

### EAE induction and treatment

EAE was induced by immunization with > 95% pure synthetic MOG_35-55 _peptide (rat MOG_35-55_, MEVGWYRSPFSRVVHLYRNGK; EspiKem Srl, Florence, Italy). Mice were injected subcutaneously at one side of the flank with 100 μL solution containing 150 μg of rat MOG in complete Freund's adjuvant (Sigma-Aldrich, St. Louis, MO, USA) and 5 mg/mL Mycobacterium tuberculosis H37Ra (Difco Laboratories, Detroit, MI, USA). Mice also received intraperitoneal injections of 150 ng pertussis toxin (Sigma-Aldrich, St. Louis, MO, USA) in 100 μL PBS immediately after MOG injection and 48 h later. Mice were scored daily for signs of EAE on a scale of 0 to 6 using the following criteria: 0, no clinical signs; 1, distal limp tail; 1.5, complete limp tail; 2, mild paraparesis of the hind limbs, unsteady gait and impairment of righting reflex; 3, moderate paraparesis, partial hind limb paralysis, voluntary movements still possible and ataxia; 4, paraplegia and forelimb weakness; 5, tetraparesis; 6, moribund state. When clinical signs were intermediate between two grades of the disease, 0.5 was added to the lower score. To study the effects of the drug, two different administration protocols were performed: in the first one, treatment began on the first day of EAE induction whereas the second one started when the EAE clinical score was ≥ 1 (appearance of clinical signs). The MOG-immunized mice were administered either 0.8 mg/kg diazoxide (treated group) or diluent (0.3% DMSO in water, vehicle group) for 30 or 15 days by oral gavage, respectively.

### Blood glucose measurements

Blood glucose measurements were performed using an Accu-Chek^® ^Aviva glucometer (Roche Diagnostics, Basel, Switzerland). Blood samples were obtained from a small incision made at the distal part of the mice tail. Blood glucose concentrations higher than 176 mg/dL were considered hyperglycemic, according to animal welfare guidelines.

### Histological and immunohistochemical analysis

To analyze the efficacy of diazoxide during the chronic effector phase of EAE, histological spinal cord analysis was performed in animals treated from the appearance of the first clinical signs. At the end of treatment, animals were anesthetized, transcardially perfused with 0.01 M PBS, followed by 4% paraformaldehyde solution. Spinal cords were then collected and post-fixed in fresh fixative solution for 4 h. For cryoprotection, they were placed in 30% sucrose for 24 h. Tissue was frozen in isopentane and dry ice and stored at -80°C. Coronal sections (20 μm) at the cervical, thoracic and lumbar levels were obtained in HM550 Cryostat (Thermo Scientific, Waltham, MA, USA) at -22°C and deposited onto poly-L-lysine-coated microscope slides.

Hematoxylin and eosin (H&E), Luxol fast blue (LFB), Nissl and Bielschowsky silver staining were used for histological studies.

For immunohistochemical studies, sections were first blocked in PBS (0.5% Triton) containing 10% goat serum (Sigma-Aldrich, St. Louis, MO, USA) for 2 h. The sections were then incubated with primary antibodies at 4°C overnight, followed by secondary antibodies for 2 h in blocking solution. The following antibodies were used: anti-Kir6.1 and anti-Kir6.2 (1:150 dilution, Alomone, Jerusalem, Israel), anti-CD11b and anti-CD3 (1:400 and 1:300 respectively, Serotec, Oxford, England, UK), anti-glial fibrillary acidic protein (GFAP) (1:2000, Dako, Glostrup, Denmark), anti-CD20 (1:300, Santa Cruz Biotechnology, St. Cruz, CA, USA) and anti-Neuronal nuclei (NeuN) (1:500, Millipore, Bedford, MA, USA). The secondary antibodies used were Alexa^®^488 and 596 (from 1:2000 to 1:1000, Molecular Probes, Invitrogen, Eugene, OR, USA). To assess the number of cells, the nuclear stain Hoechst 34580 (2 μg/mL; Molecular Probes, Invitrogen, Eugene, OR, USA) was added prior to final washes after secondary antibody addition. Sections were mounted using ProLong Gold antifade medium (Molecular Probes, Invitrogen, Eugene, OR, USA). As absolute controls, non-immunized healthy mice were also analyzed.

### Quantification of histology and immunohistochemistry

Images were captured using both wide field microsope Leica AF7000 (Leica Microsystems GmbH, Wetzlar, Germany) located at the Institut de Biologia Molecular de Barcelona, Microscopy Unit, Parc Científic de Barcelona, and SP1 confocal microscope. The analyses were carried out on three randomly selected sections of cervical, thoracic and lumbar spinal cord per animal (n = 4 to 8 animals/group) to assess demyelination, number of inflammatory/infiltration lesions, reactive microglial-macrophage areas, astrocytic reactivity and number of infiltrating cells. To assess axonal loss area and for neuronal counting, the thoracic region (n = 6 to 8 animals/group) was used.

The resulting area and cell measurements were quantified using ImageJ software analysis (National Institute of Health, USA). For astrocytic reactivity, after defining the threshold for background correction, the integrated density of GFAP labeling was measured. The integrated density is the area above the threshold for the mean density minus the background. All analyses were performed blind with respect to the experimental groups.

### Statistical Analysis

Data are expressed as the mean ± SEM unless specified. Statistical analysis of cell treatments was carried out using one-way ANOVA followed by Newman-Keuls post test when three or more experimental groups were compared. Data on the effect of EAE treatment on clinical signs, histological and immunohistochemical analysis were analyzed by Student's *t*-test or Mann-Whitney test for nonparametric data. Values of *p *< 0.05 were considered statistically significant.

## Results

### Expression and localization of K_ATP _channels in microglial cells

**Figure 1:**
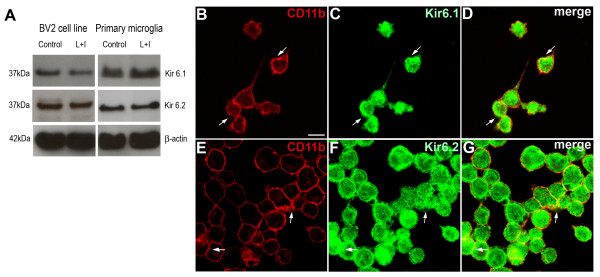
**Western blotting show expression of both Kir6.1 and Kir6.2 K_ATP _channel pore-forming subunits in unstimulated and LPS/IFNγ-stimulated BV-2 cells (A, left) and microglial primary cultures (A, Right). Staining for the microglial cell membrane marker CD11b (B and E) and the K_ATP _channel subunits Kir 6.1 (C) or Kir 6.2 (F) showed colocalization in BV-2 microglia, indicating the expression of the K_ATP _channel at the cytoplasmic membrane (D and G, white arrows)**. Control: unstimulated cells; L+I: cells stimulated with LPS and IFNγ. Scale bar = 30 μm.

To confirm the presence of pore-forming Kir (Kir6.1 and Kir6.2) subunits of K_ATP _channels we studied their expression by western blot. Unstimulated and LPS+IFNγ stimulated primary microglia cultures and BV-2 cells were analyzed. A strong signal for both subunits in all conditions was observed (Figure 1A).

To determine the subcellular distribution of K_ATP _channels in BV-2 microglial cells, double immunofluorescence against the microglial membrane marker CD11b and Kir6.1 or Kir6.2 were performed. Results showed co-localization of CD11b and both Kir6.X subunits immunosignal at membrane level as well as in the cytosol (Figure 1B-G). These findings suggest a localization of K_ATP _channels to both plasma membrane and internal cellular components.

### Diazoxide exerts an anti-inflammatory effect on microglia *in vitro *without altering phagocytic capacity

**Figure 2:**
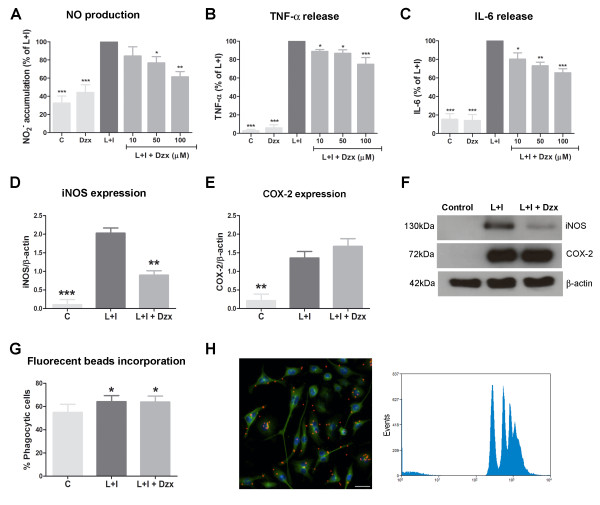
**Anti-inflammatory effects of diazoxide pre-treatment in microglial cell cultures stimulated with LPS and IFNγ**. Nitrite accumulation (A), and TNF-α (B) and IL-6 release (C) in control (unstimulated cells), DZX (unstimulated cells pretreated with 100 μM diazoxide), diazoxide (DZX) and LPS/IFNγ + Diazoxide (10 μM to 100 μM) normalized for LPS/IFNγ untreated cells. Quantification of iNOS (D) and COX-2 (E) protein expression in control, LPS/IFNγ untreated cells and 100 μM diazoxide pre-treated LPS/IFNγ cells. Protein expression was measured by western blot and data normalized with β-actin. Images showing representative immunoblotting (F). Percentage of phagocytic cells quantificated by fluorescent microspheres incorporation of control, LPS/IFNγ untreated cells and 100 μM diazoxide pre-treated LPS/IFNγ cells (G). One representative phagocytosis experiment is shown (H,left). Phagocytosis of microspheres is represented by the peaks at the high fluorescence levels (H,right) Control: unstimulated cells; DZX: unstimulated cells pretreated with 100 μM diazoxide; L+I: cells stimulated with LPS and IFNγ; L+I+DZX: L+I-stimulated cells pretreated with diazoxide. Results are shown as mean ± SEM of three to five independent experiments. *p < 0.05, **p < 0.01, ***p < 0.001 *vs *L+I for A-E and *vs *control for G.

Primary microglia cultures were used to study the ability of diazoxide to inhibit the release of inflammatory signals. Microglia activation was induced with 100 ng/mL LPS and 10 pg/mL IFNγ, and the evaluation of the inflammatory response was studied thought the measure of NO production, and TNF-α and IL-6 release in the media. Microglia cells showed an increase of NO production and cytokines release 24 h after the LPS/IFNγ stimulation. Diazoxide pre-treatment before stimulation decreased NO production (up to 38.8 ± 6.6%; Figure 2A) and TNF-α and IL-6 release (up to 25.0 ± 8.2% and 34.6 ± 5.1% respectively; Figure 2B-C) in a dose-dependent manner. Unstimulated cells treated with diazoxide 100 μM showed no differences compared to control cells (Figure 2A-C). Similar results were observed when diazoxide was tested in BV-2 microglia and primary mixed glial cultures, composed by 75% astrocytes and 25% microglia (data not shown).

Next, we evaluated iNOS and COX-2 expression in microglial cultures by western blot. Diazoxide 100 μM pre-treatment also inhibited induction of iNOS expression observed after LPS/IFNγ stimulation, while no effect on induction of COX-2 expression was detected (Figure 2D-F).

We also studied the phagocytic ability of microglia by measuring the uptake of fluorescent microspheres by BV2 cells. Stimulation with LPS/IFNγ for 24 h induced an increase in the percentage of phagocytic cells when compared to unstimulated microglia. This effect was not modified when pre-treatment with 100 μM diazoxide was performed (Figure 2G). The single-cell fluorescent population was selected on a forward-side by flow cytometry and phagocytosis of microspheres was represented by the peaks at the high fluorescent levels (Figure 2H).

### K_ATP _channel pore-forming Kir subunit expression is enhanced in activated microglia in EAE mice

**Figure 3:**
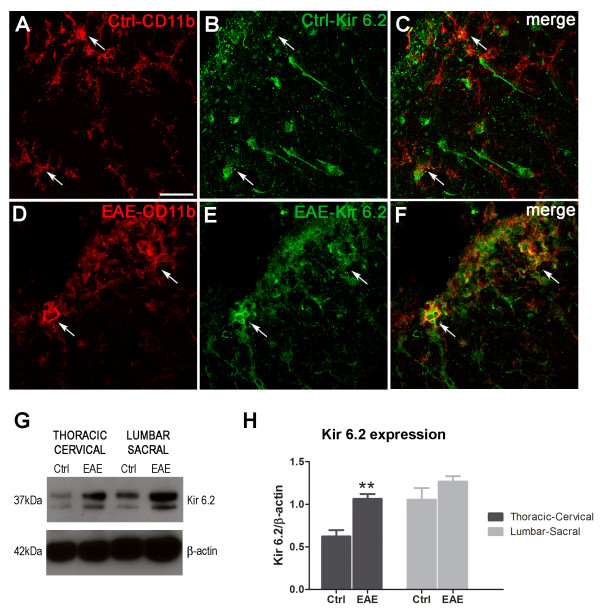
**Confocal double immunofluorescence images of CD11b (red, A and D) and Kir6.2 (green, B and E) in spinal cord sections from healthy control mice (Ctrl) or MOG_35-55 _EAE mice**. A slight intensity was found for Kir6.2 in healthy section showing low localization of the K_ATP _Kir6.2 subunit in CD11b-positive cells (white arrows, C). However, higher intensity of Kir6.2 subunit in CD11b reactive cells showing a strong colocalization of both (white arrows, F) was observed. Western blotting for Kir6.2 in total protein homogenates from lumbar-sacral and thoracic-cervical regions of the spinal cord from non-immunized control animals (control, G) and EAE mice (EAE, G) show an increase in Kir6.2 expression in EAE mice. This increase is statistically significant in the thoracic-cervical level of the spinal cord (H). Results are shown as mean ± SEM. **p < 0.01 between control and EAE. Scale bar = 30 μm.

To analyze the presence of K_ATP _pore-forming Kir components in EAE, double immunofluorescence staining against neuronal (NeuN), astrocytic (GFAP) or microglial/macrophage (CD11b) specific markers and Kir6.1 or Kir6.2 were performed. Spinal cord coronal sections from MOG_35-55_-immunized EAE mice and non-immunized healthy control animals were analyzed. Corresponding technical controls and single immunofluorescence detection were carried out for all situations. Strong Kir6.1 and Kir6.2 fluorescence signals were observed in NeuN- and GFAP-positive cells in spinal cord sections from both EAE and control mice (data not shown). Low basal levels of Kir 6.1 and Kir6.2 were discernible in CD11b-positive cells, corresponding to that in resting microglia of non-immunized control animals (Figure 3A-C). When spinal cord sections from EAE mice were analyzed, colocalization of both Kir6.x subunits and CD11b was observed in cells that displayed the characteristic amoeboid morphology of activated microglia/macrophages (Figure 3D-F for Kir6.2 and Additional File 1 Figure S1A-C for Kir6.1).

The quantities of Kir6.1 and Kir6.2 were examined by Western blotting of total protein extracted from sacro-lumbar and thoracic-cervical sections of spinal cords from EAE and non-immunized mice. When protein extracts from EAE and non-immunized healthy control animals were compared, no significant changes in the Kir6.1 immunoblotting signal were observed (Additional File 1, Figure S1D and E), whereas an increase in Kir6.2 expression was observed in the thoracic-cervical and lumbar-sacral sections of spinal cord from EAE mice, this observation was significant in the thoracic-cervical section (Figure 3G-H).

### Oral administration of diazoxide ameliorates clinical signs in EAE mice

**Figure 4:**
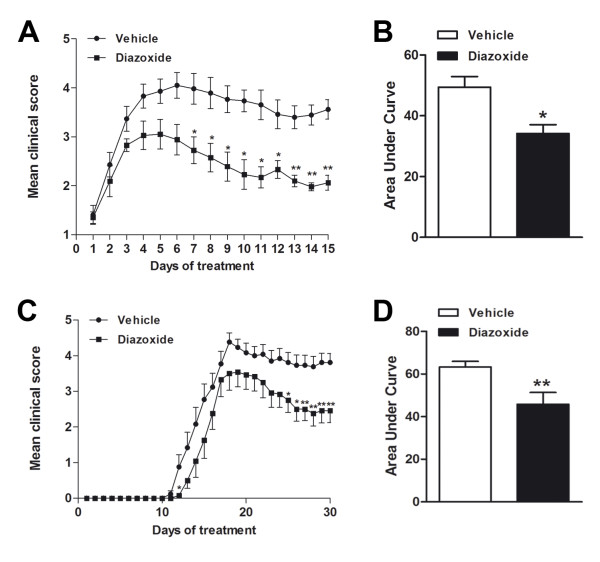
**Diazoxide treatment improves clinical signs in the EAE model**. Animals were orally administered with 0.8 mg/kg diazoxide or vehicle (0.3% DMSO in water) at the onset of clinical signs (day 10-13 post immunization) (Score ≥ 1). Once the treatment started the animals were orally treated for 15 days. A minimum of 7 mice per group was used in each experiment. Data show the mean of three independent experiments (A) and AUC for each clinical score curve (B). Diazoxide treatment was also tested for 30 days by starting its administration on the same day as that of the MOG_35-55 _immunization. Data show the daily score mean (C) and the AUC for each clinical score curve (D). Data are represented as mean ± SEM. * *p *< 0.05, ** *p *< 0.01.

MOG_35-55_-immunized mice developed severe EAE, with the onset of clinical signs occurring on days 10 to 13 after immunization. Symptoms peaked at days 13 to 16 and were followed by a stable chronic phase of the disease. To investigate the effects of diazoxide during the chronic effector phase of EAE, diazoxide treatment began at the onset of neurological symptoms (clinical score ≥ 1) of EAE mice (days 10 to 13 post immunization). When the clinical signs appeared, mice were randomly distributed into two groups and treated for 15 days with oral diazoxide (0.8 mg/kg) or vehicle (0.3% DMSO in water), respectively. The composite results of three independent experiments are summarized in Figure 4A and Table 1. Diazoxide-treated EAE mice showed an improvement in the clinical signs of the disease when compared to vehicle-treated animals (Figure 4A). The severity of the EAE clinical score was significantly reduced from the seventh day of treatment until the end of the study. In all three experiments, diazoxide-treated mice showed a lower mean EAE clinical score for the 15 days of treatment and a lower maximal mean score than vehicle-administered animals. When the area under the curve (AUC) was analyzed, a significant reduction was found in diazoxide-treated mice (63.3 ± 2.6 *vs *45.8 ± 5.6; *p *< 0.05; Figure 4B). At the end of the study, clinical examination of the animals revealed that the majority of diazoxide-treated mice presented weaknesses of the tail and hind limb, whereas most vehicle-treated mice presented severe hind limb paraparesis. In addition, some animals in the vehicle group reached the moribund state (clinical score 6), but this never occurred in the diazoxide-treated mice group.

**Table 1:** Effects of diazoxide treatment on clinical signs during the effector phase of EAE mice

	N° animals			Mean clinical score	Area under the curve (AUC)	
			
		N° EAE death	Days of treatment	Mean	Maximal grade	
**Vehicle**						
**Exp 1**	11	1	15	3.1 ± 0.1	4.1 ± 0.4	43.9 ± 4.7
**Exp 2**	8	0	15	3.3 ± 0.2	4.4 ± 0.1	48.3 ± 2.0
**Exp 3**	7	1	15	3.5 ± 0.2	4.7 ± 0.2	56.0 ± 4.4
**Diazoxide**						
**Exp 1**	11	0	15	1.9 ± 0.1***	2.7 ± 0.3*	28.3 ± 4.4*
**Exp 2**	9	0	15	2.6 ± 0.1**	3.8 ± 0.2*	37.2 ± 3.1*
**Exp 3**	7	0	15	2.6 ± 0.1***	3.5 ± 0.5*	36.9 ± 5.1*

Summary of three independent experiments. During the effector phase of EAE, 0.8 mg/kg diazoxide significantly reduced the mean global score, maximal grade and area under the curve when compared to untreated mice. Data are represented as mean ± SEM. *p < 0.05, **p < 0.01, ***p < 0.001.

Daily oral administration of diazoxide for 30 days starting from the same day as immunization was also examined. Treatment produced a significant amelioration of the EAE clinical score (Figure 4C) and global EAE severity measured as AUC (49.4 ± 3.5 *vs *34.2 ± 2.9; *p *< 0.01; Figure 4D). All animals immunized and treated with either vehicle or diazoxide developed EAE.

To test whether the dose used to treat EAE mice caused hyperglycemia, blood glucose levels were measured over a period of 30 days. Measurements were performed before oral diazoxide (0.8 mg/kg) administration (time 0) and after 30 and 60 min. No evidence of hyperglycemia was detected at any of the analyzed time points (data not shown).

### Diazoxide treatment diminishes area of demyelination and number of inflammatory lesions during the effector phase of EAE

To examine EAE-associated demyelination and cell infiltration, histopatological studies were performed with LFB and H&E staining, respectively.

**Figure 5:**
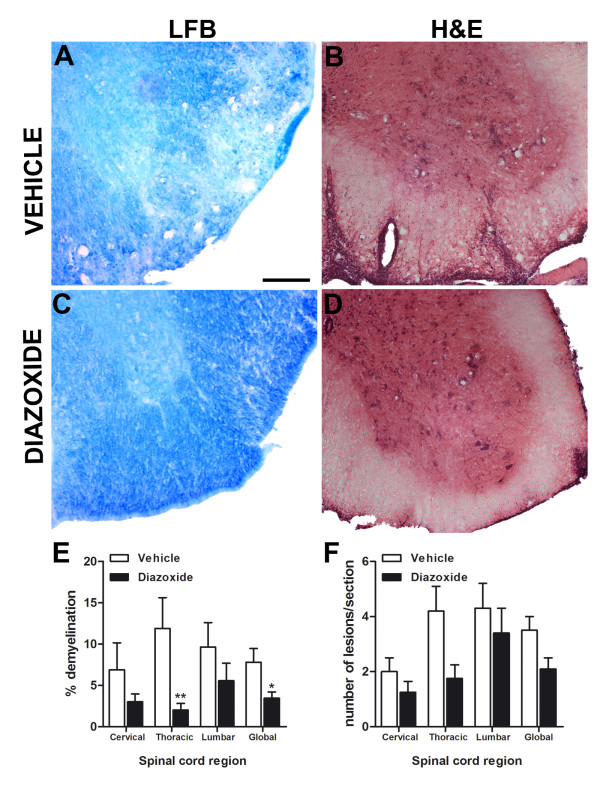
**Representative Luxol fast blue histochemical staining for myelin in coronal sections of spinal cords from vehicle- and diazoxide-treated mice (A and C, respectively)**. Quantification of the percentage of white matter area not stained by LFB shows a decrease in demyelination in diazoxide-treated mice (E). This effect was significant in the thoracic region and when the spinal cord was analyzed globally. H&E staining shows typical cell infiltrations and tissue lesions in spinal cords of vehicle- and diazoxide-treated animals (B and D, respectively). Upon quantification, results show a decrease of inflammatory lesions in all spinal cord regions (F). Results are expressed as mean ± SEM. n (animals) per group ≥ 6. Slices analyzed per animal and section ≥ 3. * *p *< 0.05, **p < 0.01. Scale bar = 100 μm.

LFB staining showed that the area of demyelination was more pronounced in the spinal cord of vehicle-treated EAE mice than in those from diazoxide-treated EAE animals (Figure 5A and 5C, respectively). The decrease in the demyelination area in diazoxide-treated mice was significant in the thoracic region and when the spinal cord was analyzed globally, compared to the vehicle-administered group (11.8 ± 3.7% *vs *2.0 ± 0.8% and 7.8 ± 2.9% *vs *3.3 ± 0.9%; *p *< 0.01 and *p *< 0.05, respectively; Figure 5E).

H&E staining of consecutive spinal cord sections of diazoxide-treated EAE mice showed a slight, but not significant, decrease in the number of lesions when compared to control EAE animals (Figure 5B, D and 5F). However, the lesions were smaller and the integrity of the tissue was better preserved in both white and gray matter in the diazoxide-administered EAE animals.

### Diazoxide treatment diminishes the astrocytic reactivity and area of activated microglia/macrophage in the effector phase of EAE

**Figure 6:**
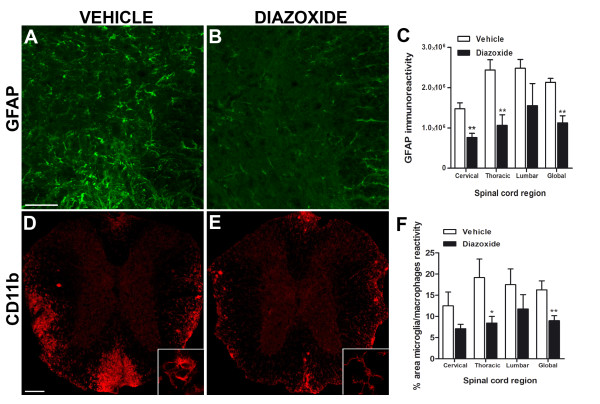
**Representative images of GFAP immunostaining of spinal cord sections from vehicle- and diazoxide-treated EAE mice (A and B, respectively)**. Upon quantification of fluorescent signal in the gray matter, results show a decrease of GFAP intensity in cervical and thoracic region and when the spinal cord was globally analyzed in diazoxide treated animals (C). CD11b immunolabeling of spinal cord sections of vehicle- and diazoxide-treated EAE mice (D and E, respectively) allows the identification of reactive microglia/macrophages in white matter as regions with ameboid-shaped cells (D bottom, image magnification) in contrast to the resting state (E bottom, image magnification). Upon quantification, results show a smaller area of reactivity in diazoxide-treated mice when compared to vehicle treated EAE mice (F). This effect was statistically significant when the thoracic level and the spinal cord was globally analyzed. Results are expressed as mean ± SEM. n (animals) per group ≥ 4 for GFAP immunoreactivity and n (animals) per group ≥ 6 for area of microglia/macrophages reactivity. Slices analyzed per animal and section ≥ 3. *p < 0.05, **p < 0.01. Scale bar = 100 μm.

To assess the consequences of diazoxide administration on astroglial reactivity, GFAP staining was performed. Results showed that the spinal cords of diazoxide-treated EAE mice exhibited less immunoreactive intensities than vehicle-treated EAE mice (Figure 6A and 6B, respectively) especially in gray matter. Fluorescent intensity quantification showed a significant decrease of GFAP signal in diazoxide treated animals in cervical (1,47.10^6 ^± 0,14. 10^6 ^*vs *0,76.10^6 ^± 0,10.10^6^;*p *< 0.01; Figure 6C) and thoracic region (2,43.10^6 ^± 0,25. 10^6 ^*vs *1,06.10^6 ^± 0,26.10^6 ^; *p *< 0.01; Figure 6C) and when the spinal cord was globally analyzed (2,13.10^6 ^± 0,09. 10^6 ^*vs *1,12.10^6 ^± 0,17. 10^6^; *p *< 0.01; Figure 6C). The classical radial morphology of GFAP-positive cells in spinal cord white matter was also better preserved in diazoxide-treated mice.

To determine the effects of diazoxide on microglial/macrophage reactivity, areas of activated CD11b-positive cells from different regions of the spinal cord were quantified. Diazoxide-treated mice showed a smaller area of reactivity than vehicle-administered EAE mice (Figure 6D and 6E, respectively). Image analysis showed a significant reduction of CD11b reactive area in the thoracic region and when the spinal cord was globally analyzed (19.1 ± 4.4% *vs *8.4 ± 1.6% and 16.25 ± 2.1% *vs *8.9 ± 1.1%; *p *< 0.05 and *p *< 0.01 respectively; Figure 6F).

### Diazoxide treatment reduces EAE-associated axonal loss and improves neuronal integrity

**Figure 7:**
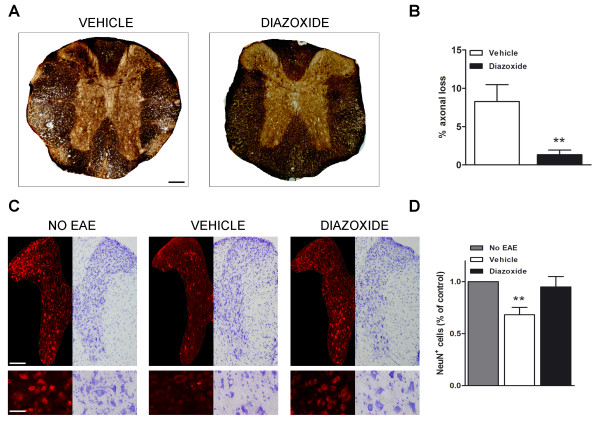
**Representative Bielschowsky stained spinal cord sections at the thoracic level of vehicle- and diazoxide-treated EAE mice (A)**. Quantification of silver staining of axons shows a decreased area of axonal loss in diazoxide-treated animals when compared to vehicle- EAE mice (B). Split image of gray matter thoracic spinal cord sections with Nissl staining and NeuN immunolabeling derived from healthy control mice (NO EAE), vehicle-treated EAE mice (VEHICLE) and 0.8 mg/kg diazoxide-treated EAE mice (DIAZOXIDE). Bottom panel shows higher magnification of neuronal integrity in the posterior (NeuN) and anterior (Nissl) section of the spinal cord (C). Upon quantification of neurons in the entire delineated gray matter, NeuN-immunolabeled neurons significantly decreased by nearly 32% in vehicle-treated EAE mice when compared to normal controls, whereas no statistically significant differences were observed between diazoxide-treated animals and normal controls (D). Results are expressed as mean ± SEM. n (animals) per group ≥ 6. Slices analyzed per animal and section ≥ 3. *p < 0.05, **p < 0.01. Scale bar = 100 μm for A,B and C (upper panel). Scale bar = 45 μm for C (bottom panel).

Bielschowsky staining was used to identify and quantify areas of axonal loss in the spinal cord of diazoxide-treated and vehicle-treated EAE mice. Diazoxide-administered EAE mice showed a significant decrease in the percentage of axonal loss when compared to vehicle-treated EAE mice (1.3 ± 0.6 *vs *8.3 ± 2.2; *p *< 0.01; Figure 7A and 7B).

To analyze the effect of diazoxide treatment on neuronal cells, NeuN immunodetection and Nissl staining were performed. NeuN immunoreactivity showed a decrease in neuronal staining in vehicle-treated EAE mice when compared to healthy controls, whereas no differences were observed between healthy animals and 0.8 mg/kg diazoxide-treated EAE mice (Figure 7C). A significant decrease (32%, *p *< 0.01) in NeuN-positive cells in gray matter at the thoracic level was found in vehicle-treated EAE mice compared to healthy mice. Diazoxide-treated animals also showed a decrease in NeuN-positive cells, but it was not significantly different from that of healthy controls (Figure 7D). Nissl staining confirmed neuronal preservation in the gray matter of diazoxide-treated mice in contrast to samples from vehicle-administered EAE mice (Figure 7C).

### Diazoxide oral treatment does not reduce lymphocytic infiltration into the spinal cord during EAE

**Figure 8:**
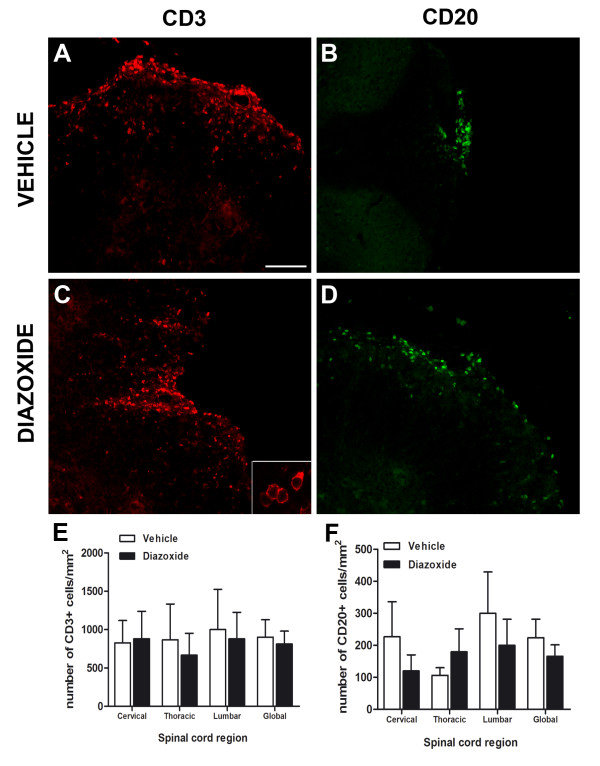
**Immunofluorescence staining for CD3 and CD20 in spinal cord slices from vehicle-administered (A and B, respectively) and diazoxide-treated EAE mice (C and D, respectively)**. Bottom panel shows higher magnification of CD3-positive cells (C). Upon quantification, no significant differences between vehicle- and diazoxide-treated mice were observed for the numbers of CD3- and CD20-positive cells in any of the spinal cord regions analyzed (E and F, respectively). Results are expressed as mean ± SEM. n (animals) per group ≥ 6. Slices analyzed per animal and section ≥ 3. Scale bar = 100 μm.

To determine whether diazoxide treatment affected T and B lymphocyte infiltration into the spinal cord of EAE mice, immunoflorescence using anti-CD3 and anti-CD20 antibodies was performed. Cell counting demonstrated no differences in the number of CD3- or CD20-immunopositive cells between vehicle-treated and diazoxide-treated animals (Figure 8A-F). A lower area of infiltration was observed in diazoxide-treated mice, corresponding to the smaller damaged areas observed in these animals.

## Discussion

K_ATP _channels are well known as linkers between cell metabolism and membrane potential. This activity has been classically described in pancreatic beta-cells, where an increase in plasma glucose promotes a calcium-dependent release of insulin due to the closing of K_ATP _channels as a result of glycolysis-mediated increases in cytoplasmic ATP levels. K_ATP _channels have also been described in the mitochondria, located on the inner membrane of these organelles where they play a crucial role in the maintenance of mitochondrial homeostasis and the proton gradient involved in the respiratory chain [30].

Besides pancreatic beta-cells, physiologically functional K_ATP _channels have been described in numerous cell types such as myocytes, neurons, astrocytes and oligodendrocytes [31-33]. In recent years, the expression of these channels in microglial cells has also been reported [17,20]. Whereas Zhou and colls. only asserted the presence of SUR2 and Kir6.1 in microglial mitochondria *in vitro*, Ramonet and colls. demonstrated the expression of SUR1 as well as SUR2 in microglia *in vivo*. In the present study, we found *in vitro *and *in vivo *inmunoreactivity for Kir6.1 in microglia and also a clear positive signal for Kir6.2. Moreover, using a specific fluorescence antibody for the K_ATP _channel Kir6.X subunits and for the microglial cell membrane marker CD11b, we found that K_ATP _channels were not restricted only to the mitochondria of BV-2 microglial cells. Our hypothesis is that microglial cells present functional K_ATP _channels at both mitochondrial and cytoplasmic membranes. Further studies are needed to analyze their functional cellular localizations in order to understand how compounds that regulate the activity of this channel affect microglial behavior.

In this way, compounds that can regulate ionic influx in microglia could represent a novel therapeutic approach for the treatment of CNS pathologies associated with microglial-mediated neuroinflammation, including EAE. In the present study, we demonstrated that diazoxide inhibited microglial inflammatory activity *in vitro*, coincidently with other authors [20,34]. Diazoxide treatment partially inhibited the inflammatory pattern induced by LPS/IFN-γ in microglial cells, inducing a decrease in NO production that could be because of the decreased expression of iNOS detected. We also observed a decrease of two major inflammatory cytokines IL-6 and TNF-α release. These pro-inflammatory agents have been shown to mediate the neurotoxic effects of reactive glial cells in vitro, and the inhibition of their production has been shown to protect against the neurotoxicity induced by reactive glial cells [35,36]. For example, expression of inducible iNOS is abundant in EAE and at the edges of MS lesions and NO is one of the main effectors of demyelination [37,38]. Microglial IL-6 secretion during EAE has been directly associated to neuronal damage [39] and leukocyte activation within the CNS [40]. TNF-α increases severity of EAE, chronic macrophage/microglial reactivity, and demyelination [41] and its inhibition prevents clinical disease despite activated T cell infiltration to the central nervous system [42] and promotes axon preservation and remyelination [43].The absence of any significant effect of diazoxide on COX-2 expression could be explained by the presence of different contributors in the final regulation of COX-2, TNF-α, IL-6 and iNOS genes under inflammatory stimuli [44,45]. Furthermore, our results showed that diazoxide had no effect on microglial phagocytosis *in vitro*. Since the clearance of debris by microglia is a primordial step for the reparative process in the spinal cord following an autoimmune attack [46,47], the maintenance of a phagocytic microglial phenotype with suppressed inflammatory behavior could be an interesting feature in demyelinating diseases. Because activated microglia (and macrophages) could exert a neuroprotective role and promote remyelination [48,49], modulation of microglia behavior would be more interesting than a total inhibition of their activation for the treatment of these diseases.

KCOs can decrease rotenone-induced mitochondrial depolarization and p38/c-Jun N-terminal kinase activation in microglia [20] by acting at the mito-K_ATP _channel level but the mechanisms involved with cytoplasmic membrane K_ATP _channels, which include changes in membrane potential and calcium influx, are yet to be elucidated. Recent studies have shown that the inhibition of N-type voltage-gated calcium channels reduced the severity of EAE neurological symptoms and decreased demyelination and infiltration areas [50,51]. The authors indicated microglia/macrophages as the principal effectors of this improvement, demonstrating that inhibition of these voltage-gated calcium channels regulates microglial activation.

Although the action of KCOs on microglia would be sufficient to explain the improvements observed in EAE mice after diazoxide treatment, the presence of functional K_ATP _channels in other glial cells and neurons could explain additional positive CNS effects induced by KCOs, especially diazoxide. In astrocytes, diazoxide exerts a neuroprotective effect by different mechanisms, including the facilitation of glutamate uptake [52] and amelioration of mitochondrial and connexin 43 dysfunction [53]. We also observed a decrease in nitrite production and inflammatory cytokines release in primary cultures that included both astrocytes and microglia and a decrease of GFAP reactivity in the gray matter of diazoxide treated EAE mice. In oligodendrocytes, diazoxide has been reported to stimulate oligodendrocyte precursor cell proliferation in a calcium-dependent manner as well as promoting myelination *in vivo *and preventing hypoxia-induced periventricular white matter injury [33]. In neurons, the positive actions of diazoxide on cell survival after cytotoxic and hypoxic/ischemic insult have been well described [19,54-57]. Moreover, a recent study in a triple transgenic mouse model of AD has demonstrated the beneficial effect of diazoxide on the improvement in cognitive tasks, reduction of anxiety, decrease in the accumulation of amyloid-beta oligomers and hyperphosphorylation of tau proteins [58]. Diazoxide may also exerts neuroprotective effects independently of K^+ ^channel activation by decreasing neuronal excitability and activation of *N*-methyl-_D_-aspartate (NMDA) receptors [18] or by increasing currents trough α-amino-3-hydroxy-5-methyl-4-isoxazolepropionic acid (AMPA) receptors [59]. The possibility of diazoxide binding to other ion channels composed by SUR subunits despite of K_ATP _channels [60,61] should not to be discarded and would need future research.

Taken together, these results reinforce our findings and could explain the differences observed between diazoxide-treated and untreated EAE mice, which included improvements in the neurological score, axon preservation and neuronal survival in addition to a decrease in glial reactivity and myelin loss.

Diazoxide-treated animals showed a decrease in disease severity a few days after the first clinical signs were observed, corresponding to the acute inflammatory phase of the disease [62]. Interestingly, we did not observe any changes in the number of infiltrating lymphocytes in the spinal cord of diazoxide-treated EAE mice when compared to vehicle-treated ones. Furthermore, the appearance of EAE signs was not prevented by diazoxide pretreatment, suggesting that oral treatment with diazoxide has no effect on the first steps of the pathology that include auto-antigen recognition, adaptive immune response and lymphocyte [63,64]. However, the effect of diazoxide on the immune system should be further explored, including direct actions of the compound on lymphocytes and peripheral macrophage populations as well as the distribution of leukocyte subpopulations during the course of EAE. Diazoxide could diminish autoimmune attacks on white matter by inhibiting microglial cells, without altering the initial immune response and infiltration regulating the pro-inflammatory environment and intercellular interactions.

## Conclusion

Daily oral administration of diazoxide in EAE mice during the effector phase of the disease reduced the severity of the clinical signs without any apparent adverse effect. Histological studies revealed that diazoxide decreased demyelination and axonal loss, reduced tissue damage, inhibited microglial/macrophage and astrocytic activation and preserved neuron integrity. No effects were observed on the number of B and T lymphocytes infiltrating the spinal cord.

We demonstrated the presence of K_ATP _channels in microglia and that its pharmacological activation produces an anti-inflammatory effect on reactive microglial cells. Diazoxide treatment of LPS and IFNγ-activated microglial cells reduced NO, IL-6 and TNF-α production as well as iNOS expression. COX-2 expression and phagocytosis showed not to be altered by diazoxide treatment.

We conclude that oral administration of diazoxide constitutes an appropriate therapeutic approach for treating MS and other demyelinating diseases involving neuroinflammation and neurodegeneration.

## List of abbreviations

AD: Alzheimer's disease; AMPA: α-amino-3-hydroxy-5-methyl-4-isoxazolepropionic acid; AUC: Area under the curve; BSA: Bovine serum albumin; CNS: Central nervous system; COX-2: Cyclooxygenase-2; DIV: Days in vitro; DMSO: Dimethyl sulfoxide; DZX: Diazoxide; EAE: Experimental autoimmune encephalomyelitis; ELISA: Enzyme-linked immunosorbent assay; FBS: Fetal bovine serum; GFAP: Glial fibrillary acidic protein; K^+^: potassium ion; K_ATP_: ATP-sensitive potassium channel; KCOs: K_ATP _channel openers; Kir: Inward-rectifying potassium channels; H&E: Hematoxylin and eosin; HRP: Horseradish peroxidase; IFNγ: Interferon gamma; IL-6: Interleukin-6; iNOS: Inducible nitric oxide synthase; LPS: Lipopolysaccharide; LFB: Luxol fast blue; Mito- K_ATP_: Mitochondrial ATP-sensitive potassium channel; MOG: Myelin oligodendrocyte glycoprotein; MS: Multiple sclerosis; MTT: 3-(4,5-Dimethyl-2-thiazolyl)-2,5-diphenyl-2H-tetrazolium bromide; NeuN: Neuronal nuclei; NMDA: *N*-methyl-_D_-aspartate: NO: Nitric oxide; PBS: Phosphate buffered saline; ROS: Reactive oxygen species; SUR: Sulfonylurea receptor; TBS: Tris-buffered saline; TNF-α: Tumor necrosis factor alpha; VGCC: Voltage-gated calcium channel.

## Competing interests

NV, JFEP, PM, APZ, MJR, NM and MP have applied for a PCT application "Diazoxide for use in the treatment of a central nervous system (CNS) autoimmune demyelinating disease" (application number PCT/EP2011/050049).

JG declares no competing interests.

## Authors' contributions

NV, JFEP and MP designed the study; NV, JFEP, PM, APZ and JG performed it; NV and JFEP analyzed the data; and NV, JFEP and MP wrote the manuscript. MJR and NM participated in the design of the study and helped draft the manuscript. All authors have read and approved the final version of the manuscript.

## Supplementary Material

Additional File 1 **Figure S1**. Confocal double immunofluorescence images of CD11b (red, A) and Kir6.1 (green, B) in spinal cord sections from MOG_35-55 _EAE mice. Colocalization of Kir6.1 subunit in CD11b reactive cells (white arrows, C) was observed. Western blotting for Kir6.1 in total protein homogenates from lumbar-sacral and thoracic-cervical regions of the spinal cord from non-immunized control animals (control, D) and EAE mice (EAE, D). Results showed no differences in Kir6.1 expression between control and EAE mice (E). Results are shown as mean ± SEM. Scale bar = 30 μm. Click here for file

## References

[B1] Lassmann H Multiple sclerosis: is there neurodegeneration independent from inflammation? J Neurol Sci 2007 259 3 6 10.1016/j.jns.2006.08.016 17367814

[B2] Block ML Zecca L Hong JS Microglia-mediated neurotoxicity: uncovering the molecular mechanisms Nat Rev Neurosci 2007 8 57 69 10.1038/nrn2038 17180163

[B3] Ransohoff RM Perry VH Microglial physiology: unique stimuli, specialized responses Annu Rev Immunol 2009 27 119 145 10.1146/annurev.immunol.021908.132528 19302036

[B4] Wulff H Zhorov BS K+ channel modulators for the treatment of neurological disorders and autoimmune diseases Chem Rev 2008 108 1744 1773 10.1021/cr078234p 18476673 PMC2714671

[B5] Ghanshani S Wulff H Miller MJ Rohm H Neben A Gutman GA Cahalan MD Chandy KG Up-regulation of the IKCa1 potassium channel during T-cell activation. Molecular mechanism and functional consequences J Biol Chem 2000 275 37137 37149 10.1074/jbc.M003941200 10961988

[B6] Wulff H Pennington M Targeting effector memory T-cells with Kv1.3 blockers Curr Opin Drug Discov Devel 2007 10 438 445 17659485

[B7] Bittner S Bobak N Herrmann AM Gobel K Meuth P Hohn KG Stenner MP Budde T Wiendl H Meuth SG Upregulation of K2P5.1 potassium channels in multiple sclerosis Ann Neurol 2010 68 58 69 10.1002/ana.22010 20582984

[B8] Clement JP Kunjilwar K Gonzalez G Schwanstecher M Panten U Aguilar-Bryan L Bryan J Association and stoichiometry of K(ATP) channel subunits Neuron 1997 18 827 838 10.1016/S0896-6273(00)80321-9 9182806

[B9] Brayden JE Functional roles of KATP channels in vascular smooth muscle Clin Exp Pharmacol Physiol 2002 29 312 316 10.1046/j.1440-1681.2002.03650.x 11985542

[B10] Ashcroft FM K(ATP) channels and insulin secretion: a key role in health and disease Biochem Soc Trans 2006 34 243 246 16545085 10.1042/BST20060243

[B11] Nichols CG KATP channels as molecular sensors of cellular metabolism Nature 2006 440 470 476 10.1038/nature04711 16554807

[B12] Busija DW Gaspar T Domoki F Katakam PV Bari F Mitochondrial-mediated suppression of ROS production upon exposure of neurons to lethal stress: mitochondrial targeted preconditioning Adv Drug Deliv Rev 2008 60 1471 1477 10.1016/j.addr.2008.03.020 18652858 PMC2612561

[B13] Xie J Duan L Qian X Huang X Ding J Hu G K(ATP) channel openers protect mesencephalic neurons against MPP+-induced cytotoxicity via inhibition of ROS production J Neurosci Res 2010 88 428 437 10.1002/jnr.22213 19746425

[B14] Zawar C Plant TD Schirra C Konnerth A Neumcke B Cell-type specific expression of ATP-sensitive potassium channels in the rat hippocampus J Physiol 1999 514 Pt 2 327 341 9852317 10.1111/j.1469-7793.1999.315ae.x PMC2269073

[B15] Levin BE Dunn-Meynell AA Routh VH Brain glucosensing and the K_ATP _channel Nat Neurosci 2001 4 459 460 11319550 10.1038/87405

[B16] Liss B Haeckel O Wildmann J Miki T Seino S Roeper J K-ATP channels promote the differential degeneration of dopaminergic midbrain neurons Nat Neurosci 2005 8 1742 1751 10.1038/nn1570 16299504

[B17] Ramonet D Rodríguez MJ Pugliese M Mahy N Putative glucosensing property in rat and human activated microglia Neurobiol Dis 2004 17 1 9 10.1016/j.nbd.2003.11.019 15350960

[B18] Goodman Y Mattson MP K+ channel openers protect hippocampal neurons against oxidative injury and amyloid beta-peptide toxicity Brain Res 1996 706 328 332 10.1016/0006-8993(95)01367-9 8822377

[B19] Roseborough G Gao D Chen L Trush MA Zhou S Williams GM Wei C The mitochondrial K-ATP channel opener, diazoxide, prevents ischemia-reperfusion injury in the rabbit spinal cord Am J Pathol 2006 168 1443 1451 10.2353/ajpath.2006.050569 16651612 PMC1606600

[B20] Zhou F Yao HH Wu JY Ding JH Sun T Hu G Opening of microglial K(ATP) channels inhibits rotenone-induced neuroinflammation J Cell Mol Med 2008 12 1559 1570 10.1111/j.1582-4934.2007.00144.x 19012619 PMC3918072

[B21] Robin E Simerabet M Hassoun SM Adamczyk S Tavernier B Vallet B Bordet R Lebuffe G Postconditioning in focal cerebral ischemia: Role of the mitochondrial ATP-dependent potassium channel Brain Res 2011 1375 137 146 21182830 10.1016/j.brainres.2010.12.054

[B22] Ashcroft FM Gribble FM New windows on the mechanism of action of K(ATP) channel openers Trends Pharmacol Sci 2000 21 439 445 10.1016/S0165-6147(00)01563-7 11121575

[B23] Bergsten P Gylfe E Wesslen N Hellman B Diazoxide unmasks glucose inhibition of insulin release by counteracting entry of Ca2+ Am J Physiol 1988 255 E422 E427 3052102 10.1152/ajpendo.1988.255.4.E422

[B24] Petit P Loubatieres-Mariani MM Potassium channels of the insulin-secreting B cell Fundam Clin Pharmacol 1992 6 123 134 10.1111/j.1472-8206.1992.tb00103.x 1628875

[B25] Warter A Gillet B Weryha A Hagbe P Simler M Hypoglycemia due to insulinoma complicated with hepatic metastases. Excellent results after 20 months of treatment with diazoxide Ann Med Interne (Paris) 1970 121 927 934 5533592

[B26] Koch-Weser J Diazoxide N Engl J Med 1976 294 1271 1273 10.1056/NEJM197606032942306 772431

[B27] Hanley PJ Mickel M Löffler M Brandt U Daut J K(ATP) channel-independent targets of diazoxide and 5-hydroxydecanoate in the heart J Physiol 2002 542 735 41 10.1113/jphysiol.2002.023960 12154175 PMC2290447

[B28] Minners J Lacerda L Yellon DM Opie LH McLeod CJ Sack MN Diazoxide-induced respiratory inhibition - a putative mitochondrial K(ATP) channel independent mechanism of pharmacological preconditioning Mol Cell Biochem 2007 294 11 8 10.1007/s11010-005-9066-6 17136444

[B29] Saura J Tusell JM Serratosa J High-yield isolation of murine microglia by mild trypsinization Glia 2003 44 183 189 10.1002/glia.10274 14603460

[B30] Holmuhamedov EL Jahangir A Oberlin A Komarov A Colombini M Terzic A Potassium channel openers are uncoupling protonophores: implication in cardioprotection FEBS Lett 2004 568 167 170 10.1016/j.febslet.2004.05.031 15196941

[B31] Noma A ATP-regulated K+ channels in cardiac muscle Nature 1983 305 147 148 10.1038/305147a0 6310409

[B32] Thomzig A Laube G Pruss H Veh RW Pore-forming subunits of K-ATP channels, Kir6.1 and Kir6.2, display prominent differences in regional and cellular distribution in the rat brain J Comp Neurol 2005 484 313 330 10.1002/cne.20469 15739238

[B33] Fogal B McClaskey C Yan S Yan H Rivkees SA Diazoxide promotes oligodendrocyte precursor cell proliferation and myelination PLoS One 2010 5 e10906 10.1371/journal.pone.0010906 20531945 PMC2878350

[B34] Liu X Wu JY Zhou F Sun XL Yao HH Yang Y Ding JH Hu G The regulation of rotenone-induced inflammatory factor production by ATP-sensitive potassium channel expressed in BV-2 cells Neurosci Lett 2006 394 131 135 10.1016/j.neulet.2005.10.018 16257489

[B35] Bal-Price A Brown GC Inflammatory neurodegeneration mediated by nitric oxide from activated glia-inhibiting neuronal respiration, causing glutamate release and excitotoxicity J Neurosci 2001 21 6480 91 11517237 10.1523/JNEUROSCI.21-17-06480.2001 PMC6763071

[B36] Wilms H Sievers J Rickert U Rostami-Yazdi M Mrowietz U Lucius R Dimethylfumarate inhibits microglial and astrocytic inflammation by suppressing the synthesis of nitric oxide, IL-1beta, TNF-alpha and IL-6 in an in-vitro model of brain inflammation J Neuroinflammation 2010 7 30 10.1186/1742-2094-7-30 20482831 PMC2880998

[B37] Hill KE Zollinger LV Watt HE Carlson NG Rose JW Inducible nitric oxide synthase in chronic active multiple sclerosis plaques: distribution, cellular expression and association with myelin damage J Neuroimmunol 2004 151 171 179 10.1016/j.jneuroim.2004.02.005 15145615

[B38] Tran EH Hardin-Pouzet H Verge G Owens T Astrocytes and microglia express inducible nitric oxide synthase in mice with experimental allergic encephalomyelitis J Neuroimmunol 1997 74 121 129 10.1016/S0165-5728(96)00215-9 9119964

[B39] Fisher J Mizrahi T Schori H Yoles E Levkovitch-Verbin H Haggiag S Revel M Schwartz M Increased post-traumatic survival of neurons in IL-6-knockout mice on a background of EAE susceptibility J Neuroimmunol 2001 119 1 9 10.1016/S0165-5728(01)00342-3 11525794

[B40] Quintana A Muller M Frausto RF Ramos R Getts DR Sanz E Hofer MJ Krauthausen M King NJ Hidalgo J Campbell IL Site-specific production of IL-6 in the central nervous system retargets and enhances the inflammatory response in experimental autoimmune encephalomyelitis J Immunol 2009 183 2079 2088 10.4049/jimmunol.0900242 19597000

[B41] Taupin V Renno T Bourbonniere L Peterson AC Rodriguez M Owens T Increased severity of experimental autoimmune encephalomyelitis, chronic macrophage/microglial reactivity, and demyelination in transgenic mice producing tumor necrosis factor-alpha in the central nervous system Eur J Immunol 1997 27 905 913 10.1002/eji.1830270416 9130643

[B42] Korner H Lemckert FA Chaudhri G Etteldorf S Sedgwick JD Tumor necrosis factor blockade in actively induced experimental autoimmune encephalomyelitis prevents clinical disease despite activated T cell infiltration to the central nervous system Eur J Immunol 1997 27 1973 1981 10.1002/eji.1830270822 9295034

[B43] Brambilla R Ashbaugh JJ Magliozzi R Dellarole A Karmally S Szymkowski DE Bethea JR Inhibition of soluble tumour necrosis factor is therapeutic in experimental autoimmune encephalomyelitis and promotes axon preservation and remyelination Brain 2011 134 2736 2754 10.1093/brain/awr199 21908877 PMC3170538

[B44] Saha RN Pahan K Regulation of inducible nitric oxide synthase gene in glial cells Antioxid Redox Signal 2006 8 929 947 10.1089/ars.2006.8.929 16771683 PMC1963415

[B45] Tsatsanis C Androulidaki A Venihaki M Margioris AN Signalling networks regulating cyclooxygenase-2 Int J Biochem Cell Biol 2006 38 1654 1661 10.1016/j.biocel.2006.03.021 16713323

[B46] Takahashi K Prinz M Stagi M Chechneva O Neumann H TREM2-transduced myeloid precursors mediate nervous tissue debris clearance and facilitate recovery in an animal model of multiple sclerosis PLoS Med 2007 4 e124 10.1371/journal.pmed.0040124 17425404 PMC1851623

[B47] Bogie JF Stinissen P Hellings N Hendriks JJ Myelin-phagocytosing macrophages modulate autoreactive T cell proliferation J Neuroinflammation 2011 8 85 10.1186/1742-2094-8-85 21781347 PMC3149992

[B48] Butovsky O Ziv Y Schwartz A Landa G Talpalar AE Pluchino S Martino G Schwartz M Microglia activated by IL-4 or IFN-gamma differentially induce neurogenesis and oligodendrogenesis from adult stem/progenitor cells Mol Cell Neurosci 2006 31 149 160 10.1016/j.mcn.2005.10.006 16297637

[B49] Doring A Yong VW The good, the bad and the ugly. Macrophages/microglia with a focus on myelin repair Front Biosci (Schol Ed) 2011 3 846 856 10.2741/191 21622236

[B50] Tokuhara N Namiki K Uesugi M Miyamoto C Ohgoh M Ido K Yoshinaga T Yamauchi T Kuromitsu J Kimura S Miyamoto N Kasuya Y N-type calcium channel in the pathogenesis of experimental autoimmune encephalomyelitis J Biol Chem 2010 285 33294 33306 10.1074/jbc.M109.089805 20720005 PMC2963360

[B51] Gadjanski I Boretius S Williams SK Lingor P Knoferle J Sattler MB Fairless R Hochmeister S Suhs KW Michaelis T Frahm J Storch MK Bahr M Diem R Role of n-type voltage-dependent calcium channels in autoimmune optic neuritis Ann Neurol 2009 66 81 93 10.1002/ana.21668 19670438

[B52] Sun XL Zeng XN Zhou F Dai CP Ding JH Hu G KATP channel openers facilitate glutamate uptake by GluTs in rat primary cultured astrocytes Neuropsychopharmacology 2008 33 1336 1342 10.1038/sj.npp.1301501 17609675

[B53] Zhang S Liang R Zhou F Huang X Ding JH Hu G Reversal of rotenone-induced dysfunction of astrocytic connexin43 by opening mitochondrial ATP-sensitive potassium channels Cell Mol Neurobiol 2011 31 111 117 10.1007/s10571-010-9560-6 20824494 PMC11498518

[B54] Ben Ari Y Krnjevic K Crepel V Activators of ATP-sensitive K+ channels reduce anoxic depolarization in CA3 hippocampal neurons Neuroscience 1990 37 55 60 10.1016/0306-4522(90)90191-6 1978742

[B55] Nagy K Kis B Rajapakse NC Bari F Busija DW Diazoxide preconditioning protects against neuronal cell death by attenuation of oxidative stress upon glutamate stimulation J Neurosci Res 2004 76 697 704 10.1002/jnr.20120 15139028

[B56] Raval AP Dave KR DeFazio RA Perez-Pinzon MA EpsilonPKC phosphorylates the mitochondrial K(+) (ATP) channel during induction of ischemic preconditioning in the rat hippocampus Brain Res 2007 1184 345 353 17988655 10.1016/j.brainres.2007.09.073 PMC2577914

[B57] Wang L Zhu QL Wang GZ Deng TZ Chen R Liu MH Wang SW The protective roles of mitochondrial ATP-sensitive potassium channels during hypoxia-ischemia-reperfusion in brain Neurosci Lett 2011 491 63 67 10.1016/j.neulet.2010.12.065 21215294

[B58] Liu D Pitta M Lee JH Ray B Lahiri DK Furukawa K Mughal M Jiang H Villarreal J Cutler RG Greig NH Mattson MP The KATP channel activator diazoxide ameliorates amyloid-beta and tau pathologies and improves memory in the 3xTgAD mouse model of Alzheimer's disease J Alzheimers Dis 2010 22 443 457 20847430 10.3233/JAD-2010-101017 PMC2988870

[B59] Bertolino M Baraldi M Parenti C Braghiroli D DiBella M Vicini S Costa E Modulation of AMPA/kainate receptors by analogues of diazoxide and cyclothiazide in thin slices of rat hippocampus Receptor Channels 1993 1 267 78 7915948

[B60] Ammala C Moorhouse A Gribble F Ashfield R Proks P Smith PA Sakura H Coles B Ashcroft SJ Ashcroft FM Promiscuous coupling between the sulphonylurea receptor and inwardly rectifying potassium channels Nature 1996 379 545 548 10.1038/379545a0 8596634

[B61] Chen M Dong Y Simard JM Functional coupling between sulfonylurea receptor type 1 and a nonselective cation channel in reactive astrocytes from adult rat brain J Neurosci 2003 23 8568 8577 13679426 10.1523/JNEUROSCI.23-24-08568.2003 PMC6740373

[B62] Gold R Linington C Lassmann H Understanding pathogenesis and therapy of multiple sclerosis via animal models: 70 years of merits and culprits in experimental autoimmune encephalomyelitis research Brain 2006 129 1953 71 10.1093/brain/awl075 16632554

[B63] Juedes AE Ruddle NH Resident and infiltrating central nervous system APCs regulate the emergence and resolution of experimental autoimmune encephalomyelitis J Immunol 2001 166 5168 5175 11290800 10.4049/jimmunol.166.8.5168

[B64] Korner H Riminton DS Strickland DH Lemckert FA Pollard JD Sedgwick JD Critical points of tumor necrosis factor action in central nervous system autoimmune inflammation defined by gene targeting J Exp Med 1997 186 1585 1590 10.1084/jem.186.9.1585 9348316 PMC2199118

