# Supplementary figures and images for: Oral administration of the KATP channel opener diazoxide ameliorates disease progression in a murine model of multiple sclerosis
Source: J Neuroinflammation. 2011 Nov 2;8:149. doi: 10.1186/1742-2094-8-149 (PMC3215935; doi:10.1186/1742-2094-8-149)

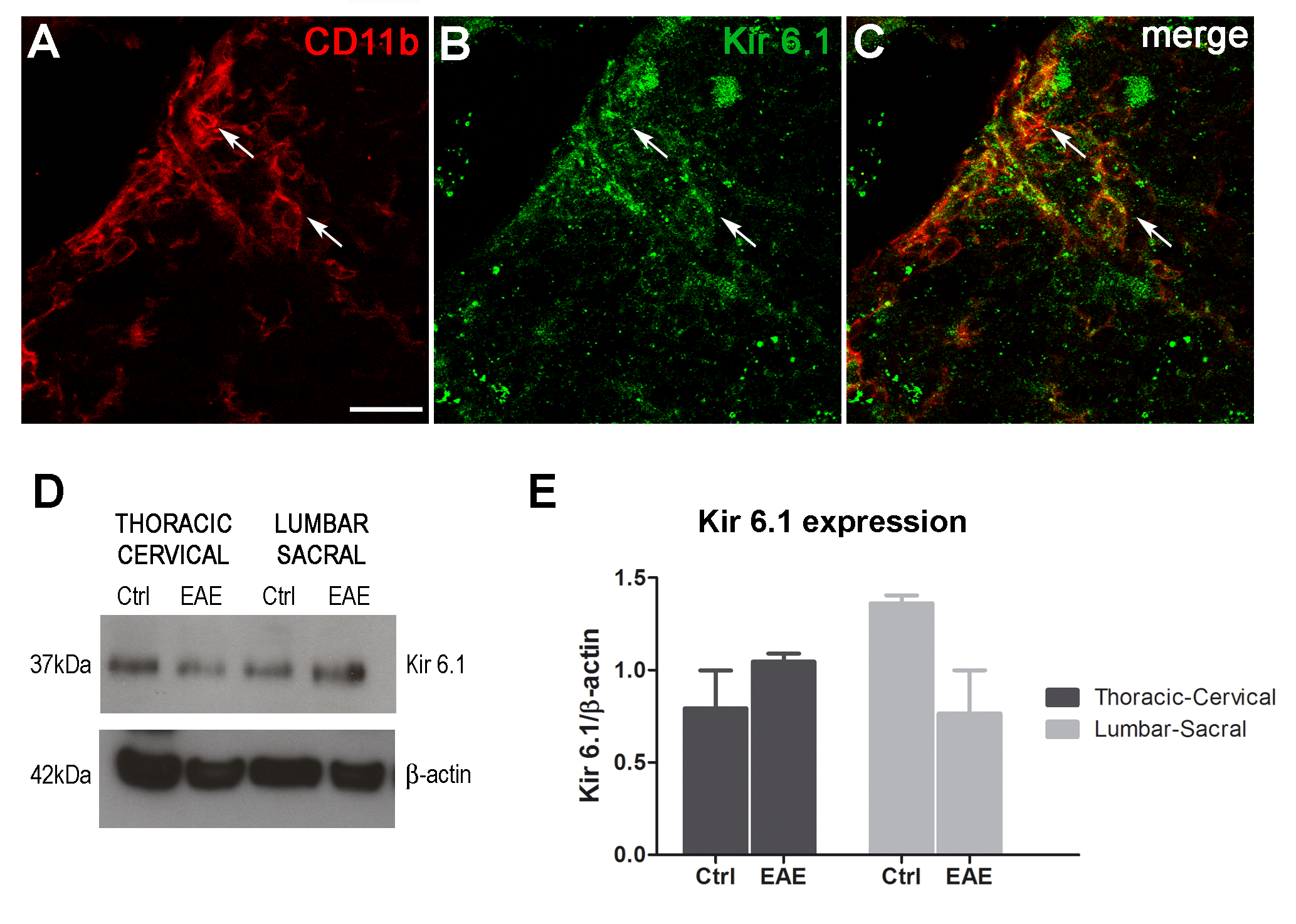

Supplement: Additional File 1 — Figure S1. Confocal double immunofluorescence images of CD11b (red, A) and Kir6.1 (green, B) in spinal cord sections from MOG35-55 EAE mice. Colocalization of Kir6.1 subunit in CD11b reactive cells (white arrows, C) was observed. Western blotting for Kir6.1 in total protein homogenates from lumbar-sacral and thoracic-cervical regions of the spinal cord from non-immunized control animals (control, D) and EAE mice (EAE, D). Results showed no differences in Kir6.1 expression between control and EAE mice (E). Results are shown as mean ± SEM. Scale bar = 30 μm. [file 1742-2094-8-149-S1.TIFF]
